# Membranoproliferative glomerulonephritis, mantle cell lymphoma infiltration, and acute kidney injury

**DOI:** 10.1007/s11255-012-0210-4

**Published:** 2012-07-01

**Authors:** Arkadiusz Lubas, Andrzej Mróz, Jerzy Smoszna, Stanisław Niemczyk

**Affiliations:** 1 Department of Internal Medicine, Nephrology and Dialysotherapy, Military Institute of Medicine, ul. Szaserów 128, 04-141 Warsaw 44, Poland; 2 Department of Gastroenterology and Hepatology, Medical Center for Postgraduate Education, ul. Roentgena 5, 02-781 Warsaw, Poland

**Keywords:** Mantle cell lymphoma, Kidney infiltration, Glomerulonephritis, Acute kidney injury

## Abstract

Mantle cell lymphoma (MCL) is a rare aggressive lymphoid neoplasm occurring in about 3–7 % of non-Hodgkin lymphomas in the United States and Europe. Although lymphomas infiltrations are recognized in about half of post-mortem studies, in available literature we found only eight cases of mantle cell lymphoma with renal involvement. Five of them present MCL related glomerulonephritis, two show renal MCL infiltration with acute kidney injury and the last one describes MCL infiltration with acute tubulo-intrerstitial nephritis. We present the first case of a patient with the coexistence of renal mantle cell lymphoma infiltration, subacute membranoproliferative glomerulonephritis and acute kidney injury.

## Case report

Patient B.C., age 59, was admitted to the Clinic of Nephrology in August 2011 with a suspected rapidly progressing glomerulonephritis. In an interview, the patient described the existence of eczema present for about 5 years, on face, chest, back and limbs recognized as rosacea-like dermatitis. So far performed diagnostics has found markers of eosinophilia increasing since 2008 (0.55–2.42 g/l) and neck lymphadenopathy (lymph nodes up to 8 × 25 mm) without further diagnostics. In 2008, the patient was temporarily treated orally with methylprednisolone and cetirizine. Additional diagnostics performed in June 2010, during another stay at the Clinic of Dermatology, discovered high concentration of total IgE (20.44 g/l), slight impairment of renal function (creatinine 106.08 μmol/l, GFR-MDRD 67 ml/min/1.73 m^2^) with proteinuria (0.5 g/l) and hematuria (16–25/high-power field) suggesting glomerulonephritis, hypercholesterolemia (6.7 mmol/l), hyperbilirubinemia (32.8 μmol/l) and elevated erythrocyte sedimentation rate (28 mm/h). In May 2011, hemorrhagic, Henoch–Schönlein-like purpura of hands, feet and legs joined the existing eczema. For this reason, in July 2011 the patient was hospitalized in the Clinic of Infectious Diseases and Allergology, where persistent features of glomerulonephritis with proteinuria 0.45 g/24 h, elevated levels of sIgA (8.37 g/l), and proper kidney function (creatinine 79.56 μmol/l, GFR-MDRD 92 ml/min/1.73 m^2^) were confirmed. A chest radiograph indicated a widened shadow of the right mediastinum. An ultrasound examination visualized numerous enlarged peripheral lymph nodes in the neck (up to 13 × 37 mm), enlarged spleen, and stones of 5–9 mm in both kidneys. A biopsy of the skin revealed erythema elevatum with neutrophilic, eosinophilic and lymphocytic infiltration around vessels. As a complication of the biopsy, erysipelas of the left upper limb occurred (August 2011), with a fever up to 39 °C, treated successfully with the second- and third-generation cephalosporins and metronidazole. During the treatment, progressive deterioration of renal function was found. At the admission to the Clinic of Nephrology, the patient did not report any essential ailments. Additional diagnostics revealed signs of progressive multi-organ failure: kidney with nephrotic syndrome (creatinine 772.92 μmol/l, GFR-MDRD 7 ml/min/1.73 m^2^; urea 27.89 mmol/l; albumin 25 g/l; proteinuria 6.88 g/24 h with preserved diuresis 2.5 l/24 h), liver (bilirubin 131.67 μmol/l, GGTP 2,938 U/l; AST 119 U/l, ALT185 U/l), and hematopoietic system (Hgb 77 g/l) with the complement system activation (C_3_ 0.23 g/l; C_4_ 0.01 g/l). The presence of ANA, p- and c-ANCA antibodies was not confirmed, but the level of dsDNA antibodies was elevated up to 44 IU/ml. Ultrasound abdomen examination showed enlargement of the kidneys (left kidney length 141 mm, right kidney 134 mm) with signs of the parenchyma swelling and bilateral kidney stones. Therefore, it was decided to initiate temporary renal replacement therapy by hemodialysis every second day, using a central venous catheter. At the same time, between hemodialysis, immunosuppressive therapy with i.v. infusions of 3 × 1.0 g methylprednisolone was started. The treatment continued with oral administration of 60 mg prednisone daily. The red blood cell concentrate was transfused. After three hemodialysis interventions, a significant improvement in renal function was achieved, and renal replacement therapy was no longer required (creatinine 114.92 μmol/l, GFR-MDRD 60 ml/min/1.73 m^2^, urea 8.3 mmol/l). Moreover, both normalization of liver function (bilirubin 13.68 μmol/l, GGTP 772 U/l, AST 16 U/l, ALT 28 U/l) and correction of anemia (Hgb 101 g/l) were achieved. A biopsy of the left kidney and a cervical lymph node was collected for histopathological evaluation. Based on the preliminary result of the renal biopsy, in which active lesions were found in the form of vascular loops necrosis and cellular crescents, intravenous infusion of 1.0 g of cyclophosphamide was administered.

**Fig. 1:**
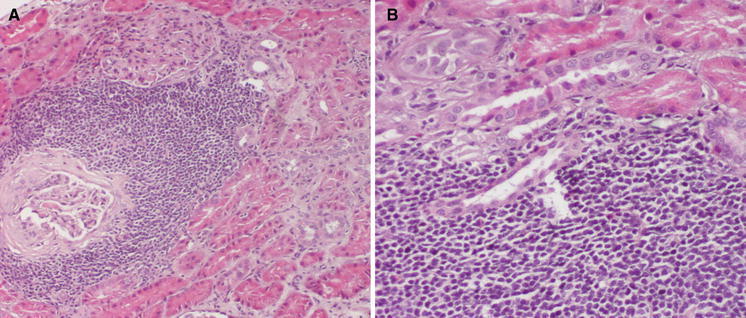
Mantle cell lymphoma infiltration in renal cortex (HE, **a** ×100, **b** ×200)

**Fig. 2:**
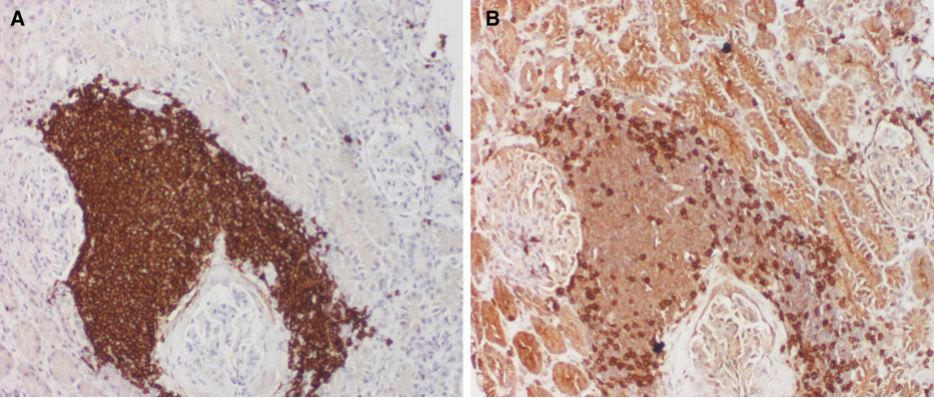
Expression of CD20 (**a** ×100) and CD3 (**b** ×100)

**Fig. 3:**
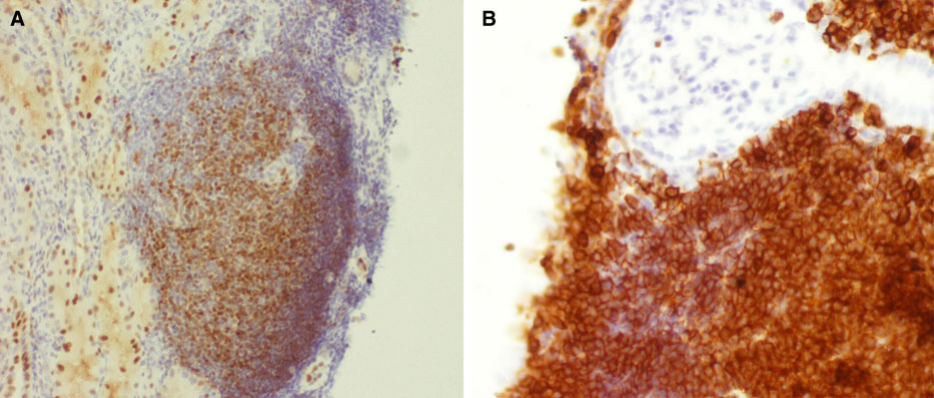
Expression of cyclin D1 (**a** ×100) and CD5 (**b** ×200)

**Fig. 4:**
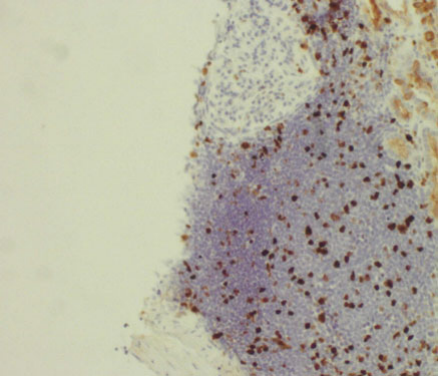
Expression of Ki-67 in about 20 % of nuclei (×100)

**Fig. 5:**
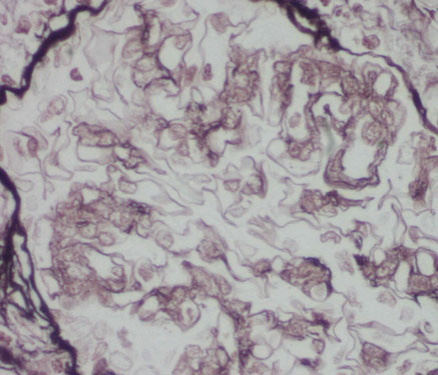
Double contouring of basal membranes and segmental sclerosis of glomerulus (×400)

Renal core biopsy submitted for embedding contained 10 glomeruli and cortex parenchyma. On histological examination, several foci of dense lymphocytic infiltration were found. These foci concentrated mainly around glomeruli and consisted of medium-sized slightly atypic lymphocytes with sparse cytoplasm. The nuclei were of similar size and cytological features and stayed in close intimacy with each other without overt molding (Fig. 1). Immunohistochemical studies revealed expression of CD20, CD5 and cyclin D1 markers, while CD3 staining was positive only in non-neoplastic cells (Figs. 2, 3). The Ki-67 staining displayed a low mitotic activity with the expression in about 20 % nuclei (Fig. 4). These morphology and immunoprofile justified the diagnosis of mantle cell lymphoma invading renal parenchyma. In addition, the biopsy showed features of membranoproliferative glomerulonephritis including mesangial proliferation and basal membranes thickening and double contouring (Fig. 5). Segmental, cellular crescents were present in 2 of 10 glomeruli as signs of glomerular sclerosis were present in several glomeruli. A striking feature of Bowman’s capsule thickening mainly in glomeruli encircled by lymphoma infiltrations was clearly visible. Immunofluorescnece staining showed C_3_ deposits in the subendothelial capillary space. In addition, only sparse mononuclear inflammatory infiltration was present within renal parenchyma as well as slight tubular injury in the form of epithelial cell edema and degeneration. The cervical lymph node biopsy confirmed the diagnosis with the recognition of mantle cell lymphoma.

The tests performed 3 weeks after leaving the hospital showed further improvement in renal function, only small-degree proteinuria with disappearance of parenchymal edema in ultrasound imaging (left kidney length 119 mm and right kidney 115 mm). Significant changes in clinical and biochemical outcomes 3 weeks after initial therapy are presented in Table 1. Patient was transferred to the Clinic of Hematology, in order to continue treatment.

**Table 1:** Significant changes in clinical and biochemical outcomes 3 weeks after initial therapy

	Before therapy	After initial therapy
Skin	Hemorrhagic, Henoch–Schönlein-like purpura	Slight (retracting) rosacea-like dermatitis
Kidney		
Function	Acute kidney injury (creatinine 772.92 μmol/l, GFR-MDRD 7 ml/min/1.73 m^2^)	Proper kidney function (creatinine 79.56 μmol/l, GFR-MDRD 92 ml/min/1.73 m^2^)
	Nephrotic syndrome (proteinuria of 6.88 g/24 h)	Small-degree proteinuria in spot morning urine sample (0.25 g/l)
Ultrasound imaging	Enlargement (left kidney—141 mm; right kidney—134 mm)	Correct length of kidneys (left kidney—119 mm; right kidney—115 mm)
	Parenchymal edema	No abnormal alterations
Liver		
Function	Acute liver injury (bilirubin 131.67 μmol/l, GGTP 2,938 U/l; AST 119 U/l, ALT 185 U/l)	Normalization (bilirubin 22.23 μmol/l; AST 19 U/l, ALT 31 U/l)
Ultrasound imaging	Hepatomegaly (length: 160 mm)	Hepatomegaly (length: 167 mm)
Spleen		
Ultrasound imaging	Splenomegaly (length: 137 mm)	Almost normal spleen length (126 mm)

## Discussion

Mantle cell lymphoma (MCL) is a rare aggressive lymphoid neoplasm occurring in about 3–7 % of non-Hodgkin (NHL) lymphomas in United States and Europe [1, 2]. Most frequent NHL infiltrating kidneys are recognized as extranodal cancers of marginal zone, especially MALT (mucose-associated lymphoid tissue lymphoma), DLBCL (diffuse large B-cell lymphoma) and Burkitt’s lymphomas [10]. Seizure of the genitourinary tract by lymphoma occurs rarely, its incidence is estimated in about 5 %, in which renal involvement is most common and occurs in approximately 37 % of cases. On the basis of retrospective studies, Da’as et al. [3] found that NHL were a rare cause of acute renal failure (9.5 %). Ultrasonographic findings are expressed as kidneys enlargement with a decreased echogenicity of the parenchyma.

Glomerulonephritis (GN), in the course of NHL is one of the causes of acute kidney injury (AKI). In most cases, membranoproliferative GN, mesangial proliferative GN, crescenic GN or minimal change GN, as well as membranous GN and IgA nephropathy are recognized, and clinically expressed as nephrotic syndrome with microhematuria, seldom with reduced complement components or cryoglobulinemia [3]. Start of chemotherapy, resulting in partial remission of NHL, correlates with the decrease in the signs of GN and improved organ function [3, 4, 9]. Infiltration of renal parenchyma by lymphoma cells is very rare and is rated in about 1 % of cases [3]. Most often, renal lymphoid infiltration is asymptomatic, but sometimes it can be a cause of acute tubulo-interstitial nephritis. Start of immunosuppressive treatment with a few 0.5–1.0 g intravenous doses of methylprednisolone administered every 1–2 days, then continued with oral prednisone, enables rapid improvement of renal function, although temporal renal replacement therapy is at times necessary [6, 7]. So far, eight cases of kidney impairment due to MCL have been described: four of them presenting proliferative GN, one focal segmental glomerulosclerosis (FSGS), two renal MCL infiltration with AKI, and the last AKI due to acute tubulo-interstitial nephritis [3–9]. The presented case is the first announcement describing the coexistence of renal infiltration by MCL with secondary membranoproliferative subacute GN and AKI. There is no clear explanation of the cause of the patient’s AKI. Renal failure occurred in the period of acute dermatitis and suggests diagnosis of post-infectious GN. Whereas participation of skin infection in the development of AKI may be important, the existence of chronic GN (at least from 2008) recognized in the interview seems to be more essential. The changes discovered in the renal biopsy showed a secondary GN with an indication of neoplastic process as the cause of the disease. On the other hand, untreated infiltration of the kidney with MCL could be the reason for their tubulo-interstitial inflammation and AKI [7]. Although renal biopsy showed only sparse mononuclear inflammatory infiltration within renal parenchyma and slight tubular injury, the examination took place 10 days after starting intravenous methylprednisolone and already achieved the returning of renal function (creatinine 97.24 μmol/l). Also, an improvement in renal function after infusions of methylprednisolone does not indicate clearly the cause of AKI with preserved diuresis. Such treatment is recommended as a part of the induction in rapidly progressing GN, acute glomerulopathies, as well as acute tubulo-interstitial renal inflammations. In the presented case, renal biopsy was performed after the improvement of renal function in order to determine diagnosis and further treatment. The diagnosis of MCL renal involvement and neoplastic generalization confirmed later in a lymph node biopsy has become the reason for systemic chemotherapy in the Clinic of Hematology.

## Summary

The presented case demonstrates for the first time the possibility of renal infiltration by MCL concomitant with secondary membranoproliferative GN. It also shows the importance of the renal biopsy as a useful diagnostic tool in case of kidney impairment due to the lymphoma.
